# Identification of novel pathogenic copy number aberrations in multiple myeloma: the Malaysian context

**DOI:** 10.1186/1755-8166-7-24

**Published:** 2014-04-01

**Authors:** Pau Ni Ivyna Bong, Ching Ching Ng, Kah Yuen Lam, Puteri Jamilatul Noor Megat Baharuddin, Kian Meng Chang, Zubaidah Zakaria

**Affiliations:** 1 Hematology Unit, Cancer Research Centre, Institute for Medical Research, Kuala Lumpur, Malaysia; 2 Institute of Biological Sciences, Faculty of Science, University of Malaya, Kuala Lumpur, Malaysia; 3 Hematology Department, Ampang Hospital, Kuala Lumpur, Malaysia

**Keywords:** Multiple myeloma, Array comparative genomic hybridization, Copy number aberrations, LYST, CLK1, ACSL1, NFKBIA

## Abstract

**Background:**

Multiple myeloma is an incurable disease. Little is known about the genetic and molecular mechanisms governing the pathogenesis of multiple myeloma. The risk of multiple myeloma predispositions varies among different ethnicities. More than 50% of myeloma cases showed normal karyotypes with conventional cytogenetic analysis due to the low mitotic activity and content of plasma cells in the bone marrow. In the present study, high resolution array comparative genomic hybridization technique was used to identify copy number aberrations in 63 multiple myeloma patients of Malaysia.

**Results:**

Copy number aberrations were identified in 100% of patients analyzed (n = 63). Common chromosomal gains were detected at regions 1q, 2q, 3p, 3q, 4q, 5q, 6q, 8q, 9q, 10q, 11q, 13q, 14q, 15q, 21q and Xq while common chromosomal losses were identified at regions 3q and 14q. There were a total of 25 and 5 genes localized within the regions of copy number gains and losses, respectively (>30% penetrance). The LYST, CLK1, ACSL1 and NFKBIA are genes localized within the copy number aberration regions and they represent novel information that has never been previously described in multiple myeloma patients.

**Conclusions:**

In general, due to the differences in genetic background, dietary and lifestyle practices of Malaysian compared to the Caucasian population, these chromosomal alterations might be unique for Asian MM patients. Genes identified in this study could be potential molecular therapeutic targets for the treatment and management of patients with multiple myeloma.

## Background

Multiple myeloma (MM) is a hematologic cancer, which is characterized by excessive numbers of malignant plasma cells in the bone marrow and over-abundance of monoclonal immunoglobulin or Bence-Jones protein (free monoclonal light chains). Globally, MM has an incidence rate of 102,000 and death rate of 72,000 people per year [1]. In Malaysia, more than 50% of myeloma patients were diagnosed at stage IV of the disease based on clinical staging system and it is more prevalent in men than women [2].

Multiple myeloma is an incurable disease and, little is known about the genetic and molecular mechanism governing its pathogenesis [3]. Genes localized to the copy number change regions are often a target of mutation and involved in tumorigenesis of hematological malignancies [4-6].

Various regions of chromosomal copy number aberrations have been described in MM including the deletions of 1p, 6q, 8p, 13q, 16q, 17p and 22q and gains of 1q, 6p, 9q, 11q, 12q, 15q, 17q, and 19q [7-12]. The most frequent chromosomal abnormality is partial or total loss of chromosome 13, which is found in approximately 50% of myeloma cases [13-17]. Apart from chromosome 13, genomic aberration of chromosome 1 is associated with a poor prognosis and it is described in up to 45% of patients with MM [18,19]. Short arm of chromosome 1 is commonly deleted, whereas the long arm tends to be amplified in MM [20].

Chromosomal abnormalities have gradually become an important prognostic factor in MM. However, more than 50% of myeloma cases showed normal karyotypes with conventional cytogenetic analysis due to the low mitotic activity and content of plasma cells in the bone marrow [21,22]. Array Comparative Genomic Hybridization (array CGH) is a more advanced molecular cytogenetic technique, which allows genome-wide screening of chromosomal alterations in a single experiment. It does not rely on metaphase spreads for analysis and is a more appropriate tool for copy number analysis of slow proliferating tumor cells such as MM.

Moreover, risk of multiple myeloma predispositions varies among different ethnicities [23]. This suggests that the genetic factors in Asian might be different from the Caucasian population. This is the first report on the copy number aberrations of Malaysian multiple myeloma patients. Information obtained in this study provides a better understanding on the chromosomal copy number changes in Asian compared to the Caucasian population.

## Results

### Copy number aberrations were found in 100% of MM patients

**Figure 1:**
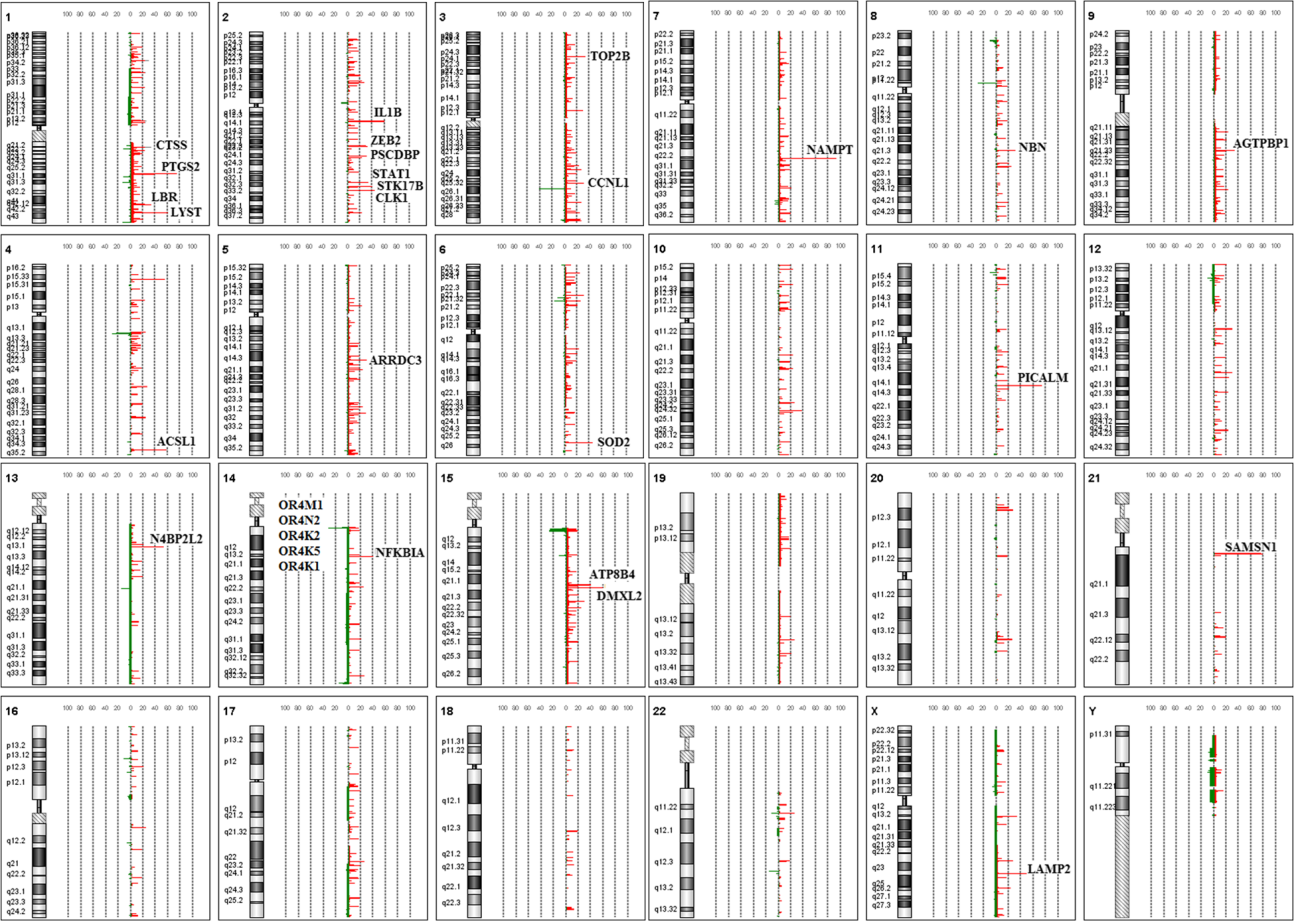
**Common copy number aberrations and percentage of penetrance in 63 MM samples analyzed.** Genes localized within the copy number aberration regions were identified (>30% penetrance). Copy number gains were indicated in red while copy number losses were indicated in green

The presence of copy number changes in 63 MM patients were analyzed by array CGH. Copy number changes were identified in 100% of the cases studied (n = 63). This frequency is consistent with the report of Largo and co-workers in 2007 [24]. The present findings showed that chromosomal gain is more common than deletion, and q-arm is more frequently altered than p-arm in the MM patients. Common chromosomal gains were identified at regions 1q, 2q, 3p, 3q, 4q, 5q, 6q, 8q, 9q, 10q, 11q, 13q, 14q, 15q, 21q and Xq while common chromosomal losses were identified at regions 3q and 14q. Figure 1 showed the common chromosomal aberrations and percentage of penetrance in all samples studied (>30%). There were 25 and 5 genes localized within the regions of copy number gains and losses, respectively (Figure 1). The aberration fragment sizes ranged from approximately 1.50 kb-0.23 Mb. An additional file shows this in more detail (see Additional file 1: Table S1).

### Copy number aberrations at chromosomes 1q42.3 and 7q22.3 were confirmed by qPCR

**Figure 2:**
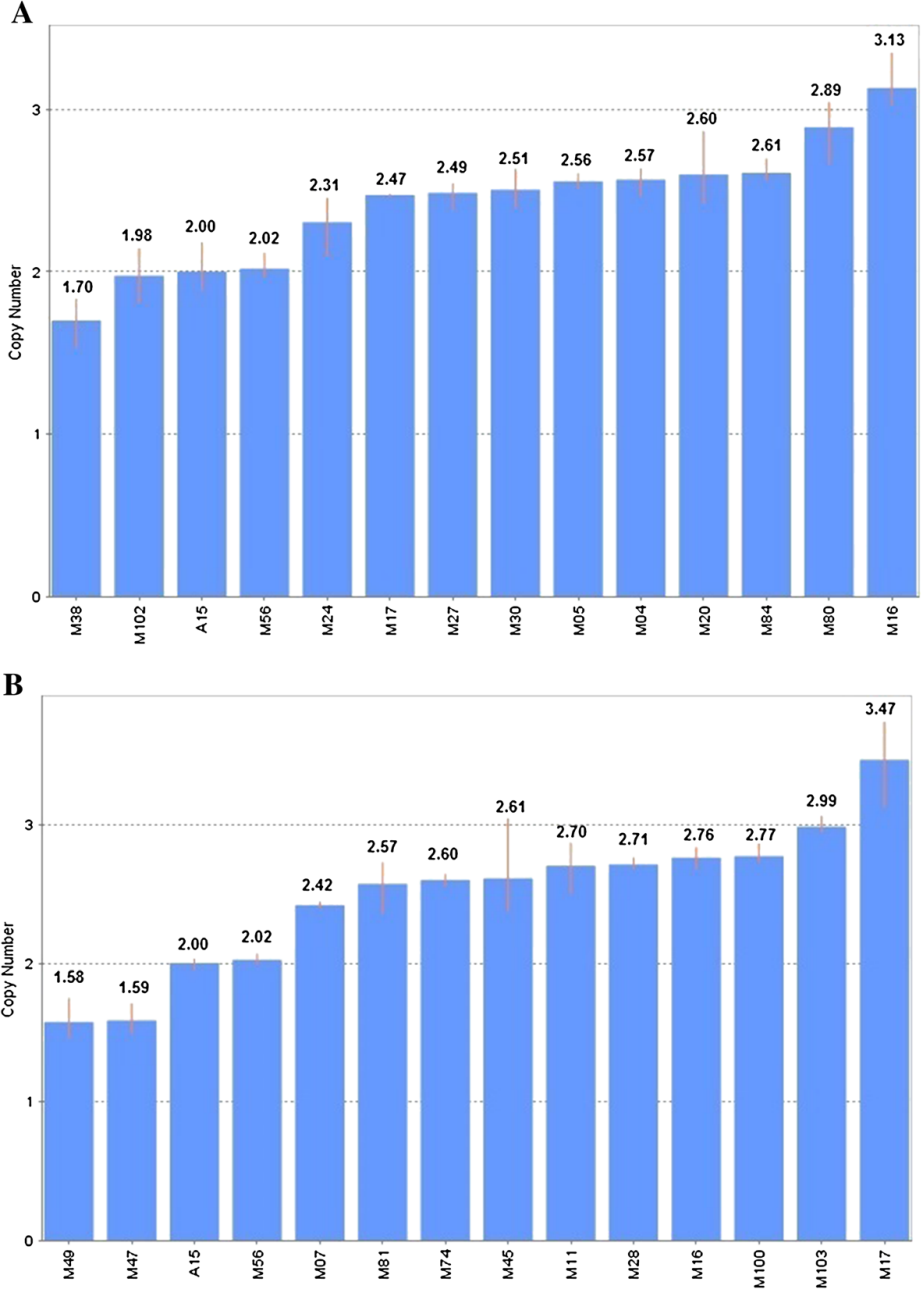
**Copy number profiles of selected genes in MM samples. (A)** Copy number profile for LYST gene, located at chromosome 1q42.3. Two copies of target were detected in negative samples (M38, M102 and M56). **(B)** Copy number profile for NAMPT gene, located at chromosome 7q22.3. Two copies of target were detected in negative samples (M47, M49 and M56). Two copies of target were detected in peripheral blood from normal individual (A15). Each sample bar represented the mean calculated copy number for three sample replicates with an error bar showing the standard deviation.

Copy number gains at chromosomes 1q42.3 and 7q22.3 were verified by TaqMan Copy Number Assay (Applied Biosystems). Nine out of 10 samples showed copy number gain at chromosome 1q42.3 except M24 which was 2.31 copies (Figure 2A). Apart from that, copy number gain at chromosome 7q22.3 was identified in 9 out of 10 samples except M07 which was 2.42 copies (Figure 2B). Although M24 and M07 were not called as significantly gains but their copy numbers were very close to 2.50. The Ct values detected by qPCR were lower in all samples if compared to the array CGH. Since the platforms and logarithms used in qPCR and array CGH were different, slight difference in calculated copy number is expected. This might explain for the lower copy numbers for M24 and M07 which did not pass the threshold set for copy number change by the software. Six samples without copy number alteration at these regions in array CGH were also negative in qPCR. They were M38, M56 and M102 for chromosome 1q42.3 and M49, M47 and M56 for chromosome 7q22.3 (Figures 2A and B).

## Discussion

Chromosomal copy number changes were identified in 100% of MM patients studied, which suggests the importance of genomic imbalances in myeloma pathogenesis. Only 2 out of 63 patients showed clonal genomic abnormalities at diagnosis by conventional G-banded karyotype analysis. These findings supported the fact that array CGH is a more powerful molecular cytogenetic technique compared to conventional G-banded karyotype analysis. Array CGH does not rely on metaphase spreads for analysis and its increased sensitivity enables the identification of relatively small amplification and deletion in the chromosome. In this study, several genes localized within the copy number change regions were revealed. These genes might represent novel candidate targets involved in the pathogenesis of MM.

The expression of the candidate genes at mRNA level has not been evaluated in this study. To further validate the copy number changes in association with gene expression, candidate genes identified in the present study were compared to the Oncomine gene expression database. Four genes localized within the copy number gain regions were significantly over-expressed at mRNA level in two published datasets on multiple myeloma patients vs normal controls (p < 0.05) [25,26]. They are NAMPT, LAMP2, SOD2 and N4BP2L2.

Nicotinamide phosphoribosyltransferase (NAMPT) is localized at chromosome 7q22.3 and this aberration region is detected in 92% of MM patients. NAMPT also known as pre-B colony enhancing factor (PBEF) or visfatin can function as a growth factor, cytokine and nicotinamide phosphoribosyltransferase [27-29]. Elevated expression of NAMPT has been implicated in MM and cancers of the prostate, brain, colon and rectum [30-33]. Over-expression of NAMPT is believed to activate nicotinamide adenine dinucleotide (NAD) salvage pathway and raise NAD^+^ level to provide sufficient energy for the survival of rapidly proliferating cancer cells. Importantly, a small molecule compound inhibitor of NAMPT, FK866 or APO866, was capable of triggering cytotoxicity in myeloma cells both *in vitro* and *in vivo*[30]. The discovery of FK866/APO866 has opened up the prospect of trailing novel targeted therapies for patients with MM. To date, FK866/APO866 has been proven as a promising anti-cancer agent in pre-clinical cancer model. Recent study shown that cytotoxic effect of FK866/APO866 is caused by autophagy rather than apoptosis [30]. Since the cytotoxic effect is activated upon successful binding between NAMPT and FK866/APO866, the utilization of FK866/APO866 as an anti-cancer agent has its limitation. For example, mutation at NAMPT binding domain inhibits FK866/APO866 from binding and consequences in drug resistance in the patient [34,35]. Therefore, new biomarkers targeting NAMPT/NAD salvage pathway are urgently needed for patients who do not benefit from FK866/APO866 therapy.

Copy number gain at chromosome segment Xq24, which coded for lysosomal associated membrane protein 2 (LAMP2) is identified in 49% of MM patients in this study. This gene functions, in part, in maintaining the integrity of the lysosomal membrane in cells [36-39]. De-regulation of LAMP2 was detected in acute myeloid leukemia (AML) [40]. Lysosomes in leukemic cells tend to be larger than those in normal cells. Thus, lysosomal mass and biogenesis are increased in AML to generate more amino acids and nucleotides for cell proliferation. Viability of leukemic cells is decreased upon knockdown of LAMP2 gene [41]. This observation suggests that elevated expression of LAMP2 might disrupt lysosomal membrane integrity, structure and size. Nevertheless, the effect of gene knockdown in MM is still uncertain. Further understanding of the functions of LAMP2 and its pathways in myeloma pathogenesis is important to elucidate whether lysosomal disruption could be a novel therapeutic strategy for the treatment of MM patients with LAMP2 over-expression and lysosomal abnormalities.

Superoxide dismutase 2 (SOD2) gene which localized at chromosome 6q25.3 was found to have copy number gain in 44% of MM cases. The SOD2 protein binds to the superoxide by products of oxidative phosphorylation and converts them to hydrogen peroxide and diatomic oxygen. Oxidative stress is an important factor in the pathogenesis of cancerous cells. The SOD2 gene expression level was decreased in tumor cells and increased in aggressive tumor [41]. Mutation in SOD2 gene has been described in breast carcinoma and mutation or methylation at the promoter region of SOD2 gene is shown to down-regulate manganese superoxide dismutase (MnSOD), a putative tumor suppressor in cancer cells [41,42]. If MnSOD is a tumor suppressor gene, tumor growth could be suppressed by restoring the expression of MnSOD in the cancerous cells. In contrast, in aggressive tumor, a high level of MnSOD is associated with NF-κB activation and this causes the decreased sensitivity of the tumor cells to chemotherapy and radiotherapy [43]. Under these circumstances, SOD2 does not act as a tumor suppressor gene. How over-expression of SOD2 impacts on tumor cells survival in advanced cancer is not well understood and needs further investigation.

On the other hand, the role of NEDD4 binding protein 2-like 2 (N4BP2L2) in the pathogenesis of MM is still unknown although it was over-expressed in MM. Nevertheless, copy number gain at chromosome 13q13.1 where N4BP2L2 is localized was found in 52% of MM patient in this study. The percentage of penetrance is quite high and this gene is worth further experimental investigation in the future.

SAMSN1 and PICALM are two potential genes localized within the copy number change regions at chromosomes 21q11.2 and 11q14.21, respectively. Both of these copy number gain regions were identified in >70% of MM cases studied. This finding is consistent with the copy number variations datasets reported by Dickens *et al.* in Oncomine database [44]. The SAMSN1 has been reported to be highly expressed in various human malignancies including myeloma, acute myeloid leukemia, and lymphoma [45,46]. It can be up-regulated when mediated by B cell activators such as IL-4, CD40L, and anti–immunoglobulin (Ig)M. The induction of SAMSN1 by IL-4 involves multiple signalling cascades including activation of Stat6, PI 3-kinase, PKC kinases, and NF-κB [45]. By knocking down SAMSN1 in lymphoma cell line did not affect the cancer cell proliferation [45]. Therefore, it is suggests that SAMSN1 is most likely participant in B cell activation and differentiation instead of proliferation. In MM, alteration in SAMSN1 might disturb the normal B cell differentiation process and eventually lead to the development of abnormal plasmablasts or plasma cells. The differentiation of B cells into immunoglobulin-secreting plasma cells is a complex process which undergoes a series of signalling pathways including extracellular signal-regulated kinase (ERK) signalling pathway. Unfortunately, the understanding of the actual mechanisms underlying the B cell differentiation process is still in its infancy. The role of SAMSN1 gene in B cell activation and differentiation and how its alteration could possibly trigger the development and clonal expansion of malignant plasma cells is a key question that remains to be investigated.

Gain of chromosome 11q14-q25 is a frequent event in MM [9,47]. The phosphatidylinositol binding clathrin assembly protein (PICALM) is localized in this region. In 2006, Largo and co-workers had reported the over-expression of the PICALM transcript and the amplification of its corresponding genomic region in MM cell lines by using microarray technology [48]. Its over-expression was confirmed in MM cell lines but not in the patient sample. The role of PICALM has been implicated in endocytosis, transcriptional regulation, iron homeostasis and cell proliferation. In addition, structural chromosomal abnormality involving genetic locus where PICALM is localized has been implicated in human hematopoietic malignancies such as leukemia and lymphomas [49-51]. Example include translocation involving PICALM and AF10 (10p12) or MLL (11q23) which resulted in fusion genes PICALM-AF10 or PICALM-MLL. PICALM rearrangements confer growth advantage by impairing endocytosis, with consequent up-regulation of its surface expression [52-55]. PICALM fusion protein has not been detected in MM. It has been shown that modulation of PICALM expression affects the intracellular iron level [56]. Therefore, it is worthy to investigate whether PICALM over-expression would enhance cell proliferation by boosting iron uptake in myeloma patients. It is important to determine whether iron chelation is a potential novel therapy for myeloma patients with high expression of PICALM and iron levels.

Information on the role of SAMSN1 and PICALM in human malignancies as discuss above had highlighted the importance of these genes in cancer genetics although there is no evidence showing that they are over-expressed at the mRNA level of MM patients. They are still worth further exploration since more than 70% of MM cases studied showed copy number gains at these regions.

**Figure 3:**
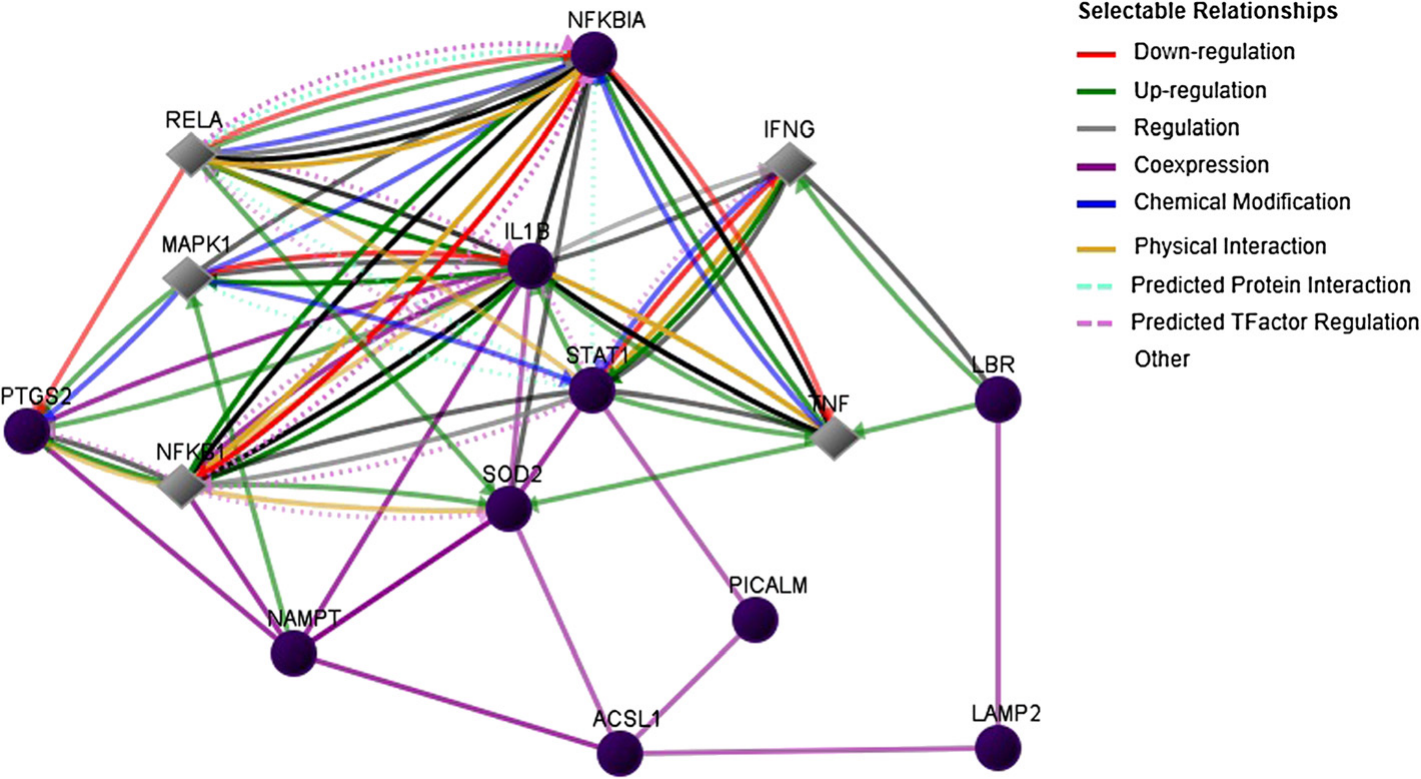
Genetic networks and pathways between genes identified by array CGH analysis.

Chromosomal segments containing genes such as LYST, CLK1, ACSL1 and NFKB1A were amplified in more than 40% of myeloma patients in this study. Information about these genes is very limited and all of them have not been previously described in association with MM pathogenesis. More studies need to be carried out to validate the function of these genes in MM predisposition. Interestingly, most of the genes identified in the common aberration regions are linked to each other via several signalling pathways such as MAPK1, NFKBI, RELA, IFNG and TNF (Figure 3).

## Conclusions

To sum up, the development of malignant plasma cells is caused by the genetic defects within the tumor and the interaction between myeloma cells and the bone marrow micro-environment. Cytotoxic resistance of myeloma cells occurred when a cascade of signalling pathways are activated upon the interaction of the cells with the bone marrow micro-environment. To avoid drug resistance and to achieve long term remission, identification of new biomarkers and novel therapeutic targets is urgently needed to improve the treatment of MM patients.

This is the first report on the identification of chromosomal abnormalities in MM patients of Malaysia. The present study generated useful information on the genomic imbalances of MM patients of Malaysia. The LYST, CLK1, ACSL1 and NFKBIA are genes localized within the copy number aberration regions and they represent novel information that have never been previously described in MM. Due to the differences in genetic background, dietary and lifestyle practices of Malaysian compared to the Caucasian population, these chromosomal alterations might be unique for Asian MM patients. Genes identified in this study could be potential molecular therapeutic targets for the treatment and management of patients with MM.

Future work aims to investigate the mRNA expression level of the genes found by array CGH using CD138+ enriched MM cells from the bone marrow. It would be of great interest to further explore the function of these genes by using *in vitro* or *in vivo* models. This will help to improve our understanding on the molecular pathogenesis of MM and eventually lead to the development of molecularly targeted therapy for treating MM patients.

## Methods

### Specimen and DNA preparation

Archival fixed bone marrow cell suspensions from cytogenetic analysis of 63 MM patients, diagnosed from year 2007-2010, were subjected for DNA isolation using the Qiagen DNA mini kit. Samples which yielded high quality and sufficient DNA were used and only newly diagnosed patients with plasma cell infiltration >10% were included in this study. The age of these patients ranged between 15-77 years with a mean and median age of 57 and 58 years, respectively. This study had been approved by the Medical Research & Ethics Committee (MREC), Ministry of Health, Malaysia. Informed consent was obtained from all patients for being included in the study. Patient characteristics are summarized in Table 1.

**Table 1:** Characteristics of 63 multiple myeloma patients

**Parameter at diagnosis**	**No. of patients (%)**
Gender	
Male	34 (53.97)
Female	29 (46.03)
Age (years)	
≤55	27 (42.86)
>55	36 (57.14)
Ethnic	
Malay	36 (57.14)
Chinese	14 (22.22)
Indian	13 (20.63)
Karyotype (G-band)	
Abnormal^a^	2 (3.17)
Normal	37 (58.73)
Unknown^b^	24 (38.10)

^a^Multiple chromosomal abnormalities were seen with karyotyping.

^b^Insufficient/ short/ no chromosome spread were available for cytogenetic analysis.

Reference DNAs were extracted from peripheral blood of healthy individuals. Only peripheral blood of individuals with normal full blood count was used. Reference DNAs were divided into 6 groups (Malay male, Malay female, Chinese male, Chinese female, Indian male and Indian female). Ten healthy individuals of specific gender and ethnicity were pooled in each group. The mean age for each reference group was as follows: Malay male, 47 years; Malay female, 34 years; Chinese male, 45 years; Chinese female, 41 years; Indian male, 48 years and Indian female, 34 years.

The quality of genomic DNA was checked on 1% denaturing agarose gel whereas purity and concentration were determined by using the NanoDrop ND-1000 UV–VIS spectrophotometer.

### Oligonucleotide array CGH

A total of 1.5 μg of Cyanine 5-dUTP-labeled test DNA and equal amount of Cyanine 3-dUTP-labeled reference DNA were mixed and used for hybridization. Reference DNAs, which matched with the patient’s gender and ethnic group were used. The mixture was then hybridized onto Human Genome CGH Microarray Kit 244 k in the presence of Cot-1 DNA (Invitrogen) for 40 h at 65°C following Agilent’s standard processing recommendations. Slides were washed (Wash Procedure B) and scanned with Agilent array scanner G2505C. Microarray images were scanned and Agilent Feature Extraction Software Version 10.7.3.1 was used to extract data from raw microarray image files in preparation for analysis.

### Data analysis

Agilent DNA Analytics software (v4.0) was used to visualize, detect and analyze aberration patterns from CGH microarray profiles. All microarray data were analyzed according to the human reference genome assembly hg18. Aberrations were detected with the ADM2 algorithm and the filtering option of a minimum of 3 probes and minimum average absolute log_2_ ratio of 0.3, with maximum aberration of 100 probes. Data were analyzed with bin size 10 and threshold 6.0. Data was filtered at a minimum of 3 probes and 30% or over for probe penetrance analysis. Common aberrations were performed with *t*-test; P threshold 0.05, overlap threshold 0.9. Aberration segments were individually reviewed with Ensembl genome browser 54. The microarray data have been submitted to the Gene Expression Omnibus (GEO) and assigned with an accession number GSE44745.

### q-PCR verification for copy number aberration

Two genomic copy number gains found by array CGH analysis were selected for verification by using TaqMan Copy Number Assay (Applied Biosystems). They were located at chromosome 1q42.3 and chromosome 7q22.3, at the molecular regions where LYST and NAMPT genes were localized, respectively. Copy number for chromosome 1q42.3 was assessed with specific TaqMan Copy Number Assay (Hs05736121_cn; Applied Biosystems). Probe and primers for chromosome 7q22.3 were custom designed as following: probe CATGATGTTACTACTTTGAAATAACC, forward primer CCTAAAGAAGATATTATCCTTGTCCTCCGTAT and reverse primer CATAGTATGCACATATTAGACTCTTCGTTGA. TaqMan Copy Number Reference Assay TERT (Applied Biosystems) was used as endogenous control.

Ten samples with copy number gains by > log_2_0.3 at chromosome segments 1q42.3 or 7q22.3 in array CGH were selected for q-PCR verification. Three MM samples with no amplification at these regions were selected as normal controls. The DNAs from normal peripheral blood designed as A15 were used as normal control in this assay. Each sample of 20 ng genomic DNA was prepared in triplicate containing TaqMan Universal Genotyping Master Mix, a FAM® dye-labeled TaqMan® Copy Number Assay and a VIC® dye-labeled TaqMan® Copy Number Reference Assay. All qPCR reactions were run on an ABI 7500 Fast Real-time PCR System (Applied Biosystems) in 96-well format and thermal cycling conditions were 95°C, 10 min followed by 40 cycles of 95°C for 15 s and 60°C for 1 min. The TERT reference gene served as endogenous control in which it is always present in two copies in a diploid genome, regardless of the copy number of the targeted of interest. It is used to normalize sample input and minimize the variation between the sample and normal control. The relative quantification analysis was then performed by CopyCaller software v1.0 (Applied Biosystems) in which 1.5 > Copy number > 2.5 will be called as having copy number change.

## Competing interests

The authors declare that they have no potential conflicts of interests.

## Authors’ contributions

IBPN was the principal investigator and takes primary responsibility for the paper; IBPN performed the laboratory work, statistical analysis, data interpretation and drafted the manuscript; NCC edited the manuscript; LKY gave technical support on statistical analysis; PJNMB and CKM gave the administrative and materials support; NCC and ZZ co-ordinated the research. All authors have read and approved the final manuscript.

## Authors’ information

IBPN is a PhD candidate and also a researcher working under Institute for Medical Research (IMR). IBPN has more than 10 years experienced in human cancer genetics research particularly in microarray and gene expression studies. NCC is an associate professor in University Malaya and she obtained her PhD in Bioscience from University of Osaka, Japan. She has more than 15 years experienced in human cancer genetics. LKY is a researcher at IMR. PJNMB is a senior researcher in IMR, she has more than 30 years experienced in leukemia research particularly specialized in molecular cytogenetics. CKM is a clinical specialist and the head of Hematology Department at Hospital Ampang and ZZ is a pathologist and head of Cancer Research Centre at the IMR.

## Supplementary Material

Additional file 1: Table S1 Summary of chromosomal copy number changes in 63 multiple myeloma patients. This table lists out all the chromosomal aberration regions identified in the current study (>30% penetrance) together with their molecular regions, percentage of penetrance and genes localized within the copy number aberration regions. Click here for file
