# Supplementary material for: Identification of novel pathogenic copy number aberrations in multiple myeloma: the Malaysian context
Source: Mol Cytogenet. 2014 Apr 1;7:24. doi: 10.1186/1755-8166-7-24 (PMC4021726; doi:10.1186/1755-8166-7-24)
Supplement: Additional file 1: Table S1 — Summary of chromosomal copy number changes in 63 multiple myeloma patients. This table lists out all the chromosomal aberration regions identified in the current study (>30% penetrance) together with their molecular regions, percentage of penetrance and genes localized within the copy number aberration regions. [file 1755-8166-7-24-S1.docx]

**Additional file 1: Table S1.** Summary of chromosomal copy number changes in 63 multiple myeloma patients

| Chromosome region | Molecular region | Aberrations | Penetrance | Gene |
| --- | --- | --- | --- | --- |
| 1q21.2 | 148971976-149005023 | Gain | 33.3 | CTSS |
| 1q31.1 | 184907807-184911998 | Gain | 38.1 | PTGS2 |
| 1q42.12 | 223656252-223669792 | Gain | 33.3 | LBR |
| 1q42.3 | 233892433-233926797 | Gain | 33.3 | LYST |
| 1q42.3 | 233926797-234063688 | Gain | 60.3 | LYST |
| 2q13 | 113303764-113310939 | Gain | 58.7 | IL1B |
| 2q22.3 | 144973952-144987741 | Gain | 31.7 | ZEB2 |
| 2q24.1 | 157979874-158003860 | Gain | 30.2 | PSCDBP |
| 2q32.2 | 191549646-191572786 | Gain | 33.3 | STAT1 |
| 2q32.3 | 196729308-196742885 | Gain | 39.7 | STK17B |
| 2q33.1 | 201430495-201434570 | Gain | 42.9 | CLK1 |
| 3p24.2 | 25625572-25659041 | Gain | 33.3 | TOP2B |
| 3q25.31 | 158349618-158359603 | Gain | 30.2 | CCNL1 |
| 4q35.1 | 185914232-185983415 | Gain | 57.1 | ACSL1 |
| 5q14.3 | 90700575-90713014 | Gain | 30.2 | ARRDC3 |
| 6q25.3 | 160023082-160033676 | Gain | 44.4 | SOD2 |
| 7q22.3 | 105685720-105700507 | Gain | 92.1 | NAMPT |
| 8q21.3 | 91015377-91052791 | Gain | 30.2 | NBN |
| 9q21.33 | 87423777-87474448 | Gain | 33.3 | AGTPBP1 |
| 11q14.21 | 85400842-85420427 | Gain | 73.0 | PICALM |
| 11q14.21 | 85420427-85452836 | Gain | 71.4 | PICALM |
| 13q13.1 | 31999429-32009312 | Gain | 52.4 | N4BP2L2 |
| 14q13.2 | 34940549-34942054 | Gain | 39.7 | NFKBIA |
| 15q21.2 | 47955679-48186620 | Gain | 39.7 | ATP8B4 |
| 15q21.2 | 49537775-49626995 | Gain | 58.7 | DMXL2 |
| 21q11.2 | 14794626-14815532 | Gain | 77.8 | SAMSN1 |
| 21q11.2 | 14815532-14840578 | Gain | 76.2 | SAMSN1 |
| Xq24 | 119454654-119467091 | Gain | 49.2 | LAMP2 |
| 14q11.2 | 19297526-19473980 | Loss | 31.7 | OR4M1, OR4N2, OR4K2, OR4K5, OR4K1 |
